# Draft genome of the Cuban Painted Landsnail *Polymita picta*, International Mollusc of the year 2022

**DOI:** 10.1186/s12863-025-01356-9

**Published:** 2025-09-03

**Authors:** Bernardo Reyes-Tur, Zeyuan Chen, Mario Juan Gordillo-Pérez, Alexander Ben Hamadou, Charlotte Gerheim, Carola Greve, Julia D. Sigwart

**Affiliations:** 1 Department of Biology and Geography, Faculty of Natural and Exact Sciences, Universidad de Oriente, Santiago de Cuba, Cuba; 2 Senckenberg Research Institute and Museum, Frankfurt, Germany; 3 Centre for Environmental Sciences, Hasselt University, Hasselt, Belgium

**Keywords:** Mollusca, Adaptive radiation, Gastropoda, Stylommatophora, Cuba, Conservation genomics

## Abstract

**Objective:**

The Cuban Painted Landsnail is an iconic endemic tree snail species with distinctive colourful shells used in traditional handicrafts. This species won the International Mollusc of the Year 2022 competition in an open public vote. As the competition prize, we have assembled the draft genome of this species.

**Data description:**

Genomic DNA from *Polymita picta* (Born, 1778) was sequenced using PacBio HiFi sequencing with a yield of 5.3 million reads (41.4 Gb) and an N50 of 8.1 Kb. The genome size of *P. picta* was estimated to be 2.9 Gb, and the final assembly was 1.85 Gb, with a total of 22,619 contigs and a contig N50 of 124.2 Kb. BUSCO analysis of the genome assembly indicated a genome completeness of 88.4%, with 7% complete duplicated BUSCOs in metazoa_odb10. The draft genome will be a valuable resource for work on the endangered Cuban Painted Landsnail including monitoring genetic diversity and establishing captive breeding for conservation.

## Objective

The Cuban Painted Landsnail, *Polymita picta* (Born, 1778), is a visually iconic species recognised for its dramatic shell colour polymorphism [1, 2] (Table 1, Data file 1 [3]). *Polymita picta* is one member of a range-restricted genus, it is endemic to eastern Cuba, and threatened by habitat loss and illegal collection [4, 5]. This species is a focal subject for evolutionary biology, ecology, and conservation [5–8]. Despite its charisma and growing interest from both scientists and collectors [5, 6], *P. picta* remains genetically understudied with only one short DNA fragment previously published [9], and one recently published mitochondrial genome [10]. Genomic resources for land snails (Stylommatophora) remain underrepresented in molluscan genomics compared to marine bivalves and cephalopods, despite the high species richness and economic importance of land snails [11]. Many available genomes are highly fragmented drafts, limited by the large genome sizes and high repeat content characteristic of gastropods [12], which complicate assembly. A high-quality *P. picta* reference genome will provide a valuable genetic resource to support genetic diversity assessment and conservation efforts, as well as to advance comparative genomic studies of land snails, particularly in the context of speciation, adaptation, and divergence.

**Table 1:** Overview of data files/data sets

Label	Name of data file/data set	File types (file extension)	Data repository and identifier (DOI or accession number)
Data file 1	Image of the Cuban Painted Landsnail *Polymita picta*	Word (.docx)	Figshare [3]: 10.6084/m9.figshare.28790750
Data file 2	HiFi sequencing of *P. picta* and quality control	Excel (.xlsx)	Figshare [3]: 10.6084/m9.figshare.28790750
Data file 3	Genome survey of *P. picta*	Portable Document Format (.png)	Figshare [3]: 10.6084/m9.figshare.28790750
Data file 4	Genome assembly statistics at different stages of *P. picta*	Excel (.xlsx)	Figshare [3]: 10.6084/m9.figshare.28790750
Data file 5	Key statistics of the final genome assembly of *P. picta*	Excel (.xlsx)	Figshare [3]: 10.6084/m9.figshare.28790750
Data set 1	Pacbio sequence reads of *P. picta* genomic DNA	Fastq files (.fastq)	NCBI Sequence Read Archive [13]: SRX28390877 http://identifiers.org/insdc.sra:SRX28390877
Data set 2	Genome assembly of *P.picta*	Fasta file (.fna)	NCBI GenBank Database [14]: JBOZOB000000000 http://identifiers.org/nucleotide:JBOZOB000000000

The International Mollusc of the Year competition has been run annually through the Senckenberg Research Institute and Museum, Frankfurt, Germany, since 2021. In order to bring more attention to the need for increasing genomic resources for molluscs. The winning species is selected by popular vote from five finalists, and as a prize the genome of the winning species is sequenced and assembled, such as this draft for *P. picta*.

## Data description

The sample used for genome sequencing was a brown shelled individual with supernumerary spiral bands (juvenile), collected by hand from El Diamante, Maisí municipality, Guantánamo province, Cuba (Table 1, data file 1 [3]). Genome size was estimated following a propidium iodide-stained nuclei flow cytometry (FCM) protocol [15], using the same *P. picta* individual used for PacBio sequencing. Neural tissue from *Acheta domesticus* (female, 1 C = 2 Gb [16]) and chicken erythrocyte nuclei (1 C = 1.2 Gb [17]) were used as internal reference standards. Here, 1 C refers to the haploid nuclear DNA content of a genome. The genome size (1 C) of *P. picta* was estimated by averaging measurements from three replicates taken on three different days to reduce random instrumental error. Genomic DNA was extracted using a CTAB-based method [18]. We prepared one PacBio ultra-low input library including a long-range PCR amplification step using the SMRTbell^®^ gDNA Sample Amplification Kit and the SMRTbell^®^ Express Template Preparation Kit 2.0. In addition, to reduce potential PCR biases of the amplification polymerases, we prepared one further library using the KOD Xtreme™ Hot Start DNA Polymerase (Merck), optimized for amplification of long and GC-rich DNA templates. For this, we combined the buffer, dNTPs and KOD polymerase from the KOD Xtreme Hot Start DNA Polymerase Kit with the ultra-low input primers from the PacBio SMRTbell gDNA Sample Amplification Kit. Otherwise, we followed the PacBio ultra-low input protocol [19, 20]. These two ultra-low input libraries were then pooled in equal mass quantities and sequenced on a single Revio SMRTcell.

HiFi reads were called using a pipeline, which is running PacBio’s tools ccs 6.4.0 [21], actc 0.3.1 [22], samtools 1.15 [23] and DeepConsensus 1.2.0 [24]. PCR adapter sequences and duplicates were removed using lima 2.9.0 [25] and pbmarkdup 1.0.3 [26], resulting in approximately 5.3 million HiFi reads (41.4 Gb) with an N50 of 8.1 kb (Table 1, Data file 2, Data set 1 [3, 13]), providing approximately 14-fold coverage of the genome of *P. picta* that was estimated at 2.9 Gb using flow cytometry. Genome size and heterozygosity were estimated from a k-mer profile of the HiFi reads using Jellyfish 2.3.0 [27] and GenomeScope 1.0 [28] using the k-mer size k = 21, and maximum k-mer coverage of 1000 as suggested. The kmer-based genome size was estimated to be 3.06 Gb, with a heterozygosity about 2.41% (Table 1, Data file 3 [3]). The cleaned HiFi reads were assembled with hifiasm 0.16.1 [29], and Purge_haplotigs 1.1.0 was used to remove duplicated contigs from the genome [30] (Table 1, Data file 4 [3]). FCS-GX 0.5.5, was used to identify and remove contaminant sequences in the new genome [31] (Table 1, Data file 4 [3]). The final *P. picta* genome assembly consists of 22,619 contigs with a total length of 1.85 Gb, a contig N50 of 124.2 Kb (Table 1, Data file 5, Data set 2 [3, 14]). Genome completeness was assessed by BUSCO (Benchmarkding Universal Single-Copy Orthologs) 5.4.3 [32], in euk_genome_met mode using the lineage dataset metazoa_odb10 and mollusca_odb10. The results showed 88.4% and 74.9% of complete BUSCOs in metazoa_odb10 (in total 954 genes) and in mollusca_odb10 (in total 5295 genes), respectively. Among them, complete single-copy genes accounted for 81.4% and 65.0%, respectively. Fragmented genes accounted for 7.3% and 4.9%, and missing genes accounted for 4.3% and 20.2%, respectively (Table 1, Data file 5 [3]).

The draft genome presents a valuable resource for monitoring genetic diversity, breeding, resilience to climate change and conservation of the endangered Cuban Painted Landsnail.

### Limitations

The polymerase amplification process has yielded a promising amount of HiFi reads for *P. picta*; however, it also shortens the average HiFi read length, resulting in a more fragmented assembly. In addition, the relatively low sequencing coverage (14× relative to the genome size) leaves some genomic regions unassembled. Lower sequencing depths can also potentially lead to errors in kmer-based estimates of genome size. The high heterozygosity of *P. picta* further complicates the assembly process, contributing to a significant proportion of the genome representing alternative haplotypes. In addition, the discrepancy between the assembly and the estimated genome size may be due to highly repetitive sequences. Nonetheless, the assembly quality is comparable to that of other published genomes of land snails and slugs, and it now provides a valuable genetic resource for comparative genomics and the discovery of lineage-specific and adaptive genes.

## Data Availability

The data described in this Data note can be freely and openly accessed on NCBI SRA under BioProject ID PRJNA1250545 [13], NCBI GenBank under accession number JBOZOB000000000 [14], and figshare (10.6084/m9.figshare.28790750) [3]. Please see Table 1 for details and links to the data.

## Abbreviations

PacBio: Pacific Biosciences Sequel II
SMRT: Single Molecule Real Time
BUSCO: Benchmarkding Universal Single-Copy Orthologs
